# Supplementary material for: The β Isoform of Human ATP-Binding Cassette B5 Transporter, ABCB5β, Localizes to the Endoplasmic Reticulum
Source: Int J Mol Sci. 2023 Oct 31;24(21):15847. doi: 10.3390/ijms242115847 (PMC10649157; doi:10.3390/ijms242115847)
Supplement: Supplementary file 1 [file ijms-24-15847-s001.zip › ijms-2653407-supplementary.pdf]

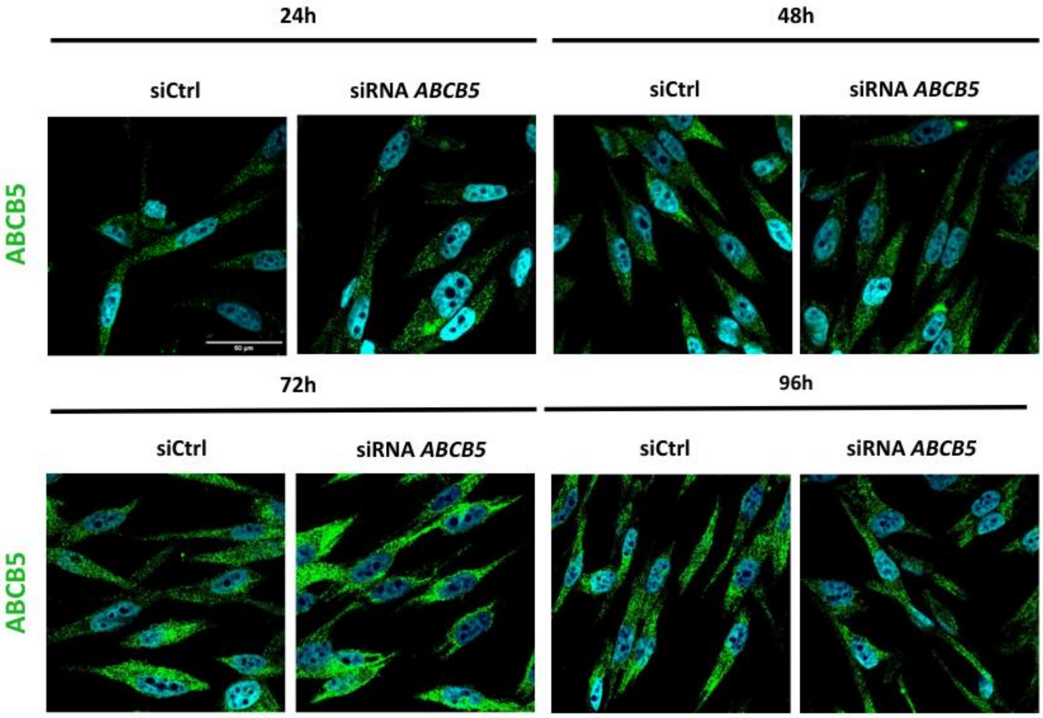

Figure S1. Immunofluorescence detection of ABCB5 in MelJuSo cells. Immunofluorescence micrographs taken at different time points after transfection of siCtrl and si*ABCB5* in MelJuSo cells. Cells were stained with anti-ABCB5 Rockland antibody followed by an AlexaFluor 488 (green) rabbit secondary antibody. Nuclei were stained with DAPI. Scale bar= 50 µm.

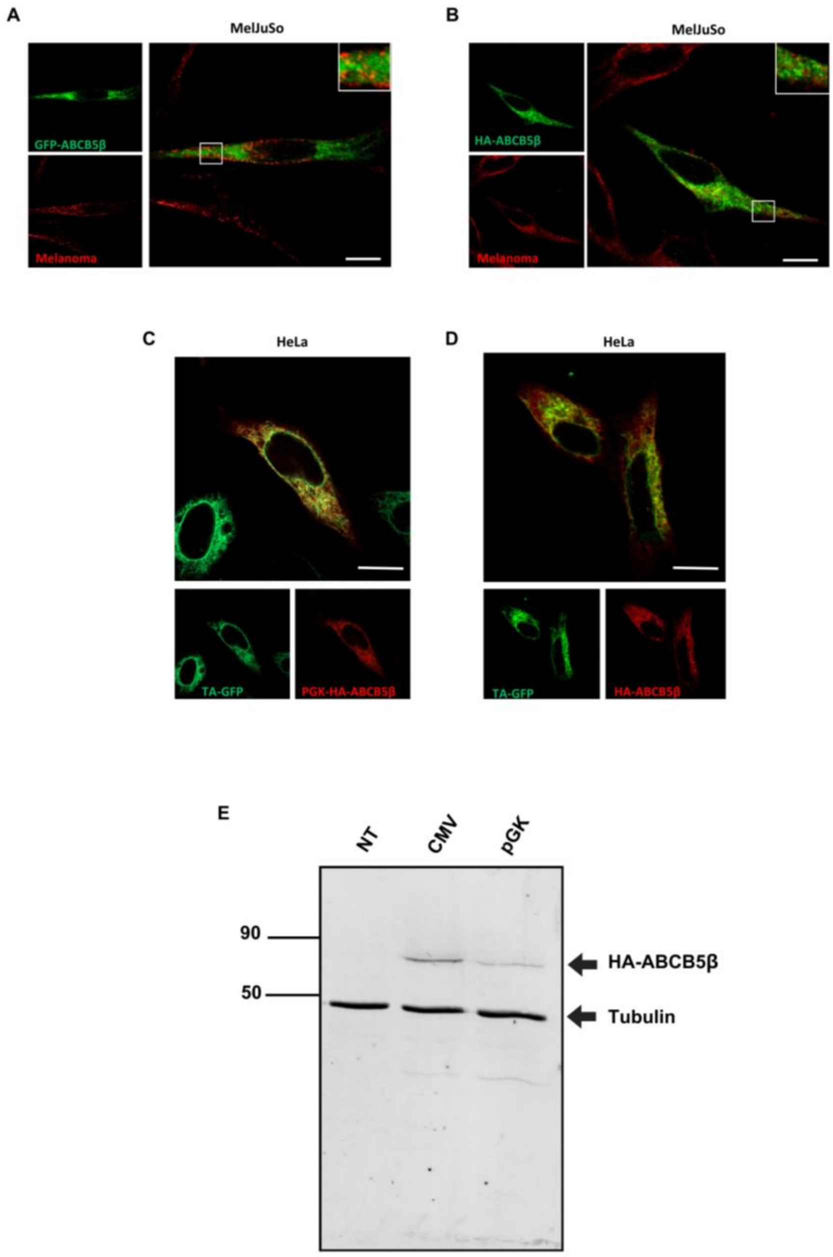

Figure S2. Complementary colocalization analyses conducted with the different ABCB5β constructs and comparison of the expression level of HA-ABCB5β when under a high (CMV) or low (pGK) expression promoter. ***A-B***, analysis of colocalization between GFP- ABCB5β **(A)** or HA-ABCB5β **(B)** with a melanoma marker expressed in MelJuSo cells. ***C-D***, Transfection of HeLa cells with either pcDNA3.1(+)-HA-ABCB5β (high expression promoter) (C), or pGK-HA-ABCB5β (low expression promoter) **(D),** and analysis of colocalization with TA-GFP (a marker of the ER membrane). (**E)** Western blotting analysis comparing the level of expression of HA-ABCB5β when under the control of the high (CMV) or low (pGK) expression promoter. NT= non transfected.
